# Trauma patients at the Helderberg District Hospital emergency centre, South Africa: A descriptive study

**DOI:** 10.1016/j.afjem.2021.03.012

**Published:** 2021-04-27

**Authors:** Trevor Marle, Robert Mash

**Affiliations:** Division of Family Medicine and Primary Care, Stellenbosch University, Box 241, Cape Town, South Africa

**Keywords:** District hospitals, Wounds and injuries, Physical trauma, Emergency care, Emergency health services, South Africa

## Abstract

**Introduction:**

Trauma is a substantial component of South Africa's burden of disease. District hospitals provide primary trauma care for a large proportion of this trauma burden, although most studies are in specialised or tertiary settings. The aim was to evaluate the profile of physical trauma patients attending the emergency centre at Helderberg District Hospital, Cape Town.

**Methods:**

An observational descriptive study was conducted between 1 January and 30 April 2019. Patients with trauma were identified from a register and systematically sampled to achieve a sample size of 377. Retrospective data from medical records was collected and analysed in the Statistical Package for Social Sciences.

**Results:**

Of the 14,873 patients attending the emergency centre 24.6% were trauma related and 381 folders were analysed. Of these patients 30.4% were female and 69.6% male with an average age of 27.8 years. Over 60% of patients used an ambulance to get to the hospital. Sundays were the busiest days with 23.9% of all cases. Intentional trauma accounted for 45.4% of cases and accidental injuries 49.1%. The commonest mechanisms were sharp injuries (27.6%), falls (22.0%) and blunt trauma (19.4%). Intentional trauma made up more than half of all trauma in males, was more prevalent than accidental trauma between 20 and 60 years and resulted in a higher proportion of admissions.

**Conclusion:**

There were high levels of intentional trauma, especially involving young males over the weekend, mostly with sharp objects. This trauma burden resulted in high numbers of admissions and transfer to tertiary hospitals. Family physicians and other generalists need to be well trained in trauma resuscitation and stabilisation. District hospital need to be appropriately equipped and supplied to manage trauma. Further research is needed to identify underlying modifiable factors that can be addressed through community-orientated interventions.

## African relevance

• District or primary hospitals run by generalists and family physicians are common in African health systems
• Such hospitals provide an important contribution to emergency medicine through their emergency centres
• Many African countries have a high burden of trauma that are managed at this level of care
• The profile of physical trauma patients managed at this level is not well described

## Introduction

Although global rates of homicide and interpersonal violence are decreasing, these remain in the top-5 causes of premature death [1]. Globally, injuries accounted for 10% of the total burden of disease [1]. More than 90% of these deaths, however, occurred in low- and middle- income countries [2]. Despite a reduction in political conflict post-apartheid, interpersonal violence has increased in South Africa over the past 20 years, resulting in this middle-income country being one of the few places where the proportion of intentional trauma is higher than accidental injury [3].

Twenty six years post democracy, South Africa is challenged by a quadruple burden of disease [4,5]. While the scourge of HIV/AIDS and tuberculosis has been most studied, there exists a substantial morbidity and mortality burden as a consequence of injury-related disorders, fuelled by alcohol and drug abuse [6], rapid urbanisation, unemployment and poverty [7]. Maternal and perinatal conditions and non-communicable diseases make up the other quadrants [4].

Homicide is consistently the leading cause of unnatural deaths in South Africa, accounting for 36% [7,8]. In males the homicide rates peak in the 15–29 year age group at 184 per 100,000, nine times the global rate [2]. In females they peak in the 30–44 age group at 32 per 100,000, seven times the global rate [2]. Traffic accidents are also significant causes of mortality and include a high proportion of pedestrians [7].

Trauma also results in long term impairments, both physical, behavioural and psychological [9,10]. This has implications for ongoing care in the health system as well as for impoverishment of families as patients are usually of working age [8,11]. The loss of productivity due to death and disability from injury represents a significant loss of economic opportunity in all countries. The treatment and rehabilitation of injured persons account for a large proportion of many national health budgets [12]. Overall, these injuries are an expensive burden on an already constrained health system due to inpatient costs, surgery, investigations and resources such as blood products [13].

In South Africa, district hospitals are mostly run by family physicians and other generalists with 24-hour emergency centres (EC). Specialised trauma and orthopaedic services are usually at the regional or tertiary levels, although some large urban district hospitals now offer this [14]. District hospitals often provide primary care for injury as clinics are closed after hours and trauma often requires expertise not available in primary care facilities. Patients requiring further medical attention are referred to regional or tertiary hospitals in order to receive specialist care, surgery, intensive care, or special investigations [15].

Until now, most research has been conducted at the tertiary level hospitals [16]. However, the bulk of all trauma is managed in regional and district level facilities, where emergency centres may not be resourced sufficiently to deal with the trauma workload [7]. Currently, very little evidence exists regarding the trauma burden at district hospitals, although two studies highlight the high prevalence of intentional injury, a male predominance and a peak of trauma on weekend nights [17,18]. Evidence on trauma in the Western Cape, at district level, is limited [19,20].

Such evidence can help identify priorities, assist in resource planning and guide the development of interventions to prevent the commonest types of trauma. This can contribute to the goal of a health system that is accessible, cost-effective and of high quality [5,9]. In addition, information could guide policy on emergency medical services and personnel training, as well as adequate staffing of emergency centres. Optimizing the transfer of patients, provision of appropriate equipment, staffing, and other resources at the correct levels of care should also result in overall cost savings [21].

The aim of the study was to evaluate the profile of trauma patients attending the emergency centre (EC) at Helderberg Hospital, Cape Town. More specific objectives included the prevalence of trauma in the EC, type of trauma, demographics of patients, temporality of trauma over the week, mode of transport to the EC and final disposition.

## Methods

### Study design

An observational descriptive study collecting retrospective data from medical records.

### Setting

Helderberg Hospital is a 181-bed district hospital situated in Cape Town. The hospital drains nine clinics and community health centres, but is the only 24-hour facility for the 600,000 population. The population is diverse and includes different socio-economic and ethnic groups, as well as urban and rural settings.

The EC is one of the busiest in the metropole with 3500 patients presenting each month. The lead clinician is a family physician. At any one time there are no more than five doctors at the EC, with four examination beds and two beds in the resuscitation unit. They are supported by two trauma sisters, working 12-hour shifts, along with other nursing and administrative staff, security and cleaners. Tygerberg hospital is the referral, tertiary level, hospital supporting Helderberg Hospital and is about 40 km away.

### Study population

All patients presenting to Helderberg Hospital with physical trauma between the 1 January and 30 April 2019 were eligible for inclusion. This included all types and mechanisms of trauma, both male and female patients, of all ages. There were no exclusion criteria.

Physical trauma was defined as injury or damage caused by exposure to physical agents (i.e. mechanical energy, heat, electricity, chemicals, or iodizing radiation) interacting with the body in amounts or at rates that exceed the threshold of human tolerance [12].

### Sample size and sampling strategy

Sample size was based on an estimated 20% proportion of patients attending the EC with trauma, a margin of error of 5%, 95% confidence intervals, and an estimated annual number of trauma patient of at least 20,000. This yielded a sample size of 377 patients. Systematic sampling of every ninth patient in the attendance register with physical trauma was used to yield a sample of at least 400 patients. In the event that a record was not found, the next record was sampled using the same systematic approach.

### Data collection

Retrospective data from the patients' notes was recorded by the researcher in a structured data collection sheet. Data was extracted on the patient's demographics (age, gender and geographical location), triage time and date, type and mechanism of trauma, use of emergency transport, triage colour and disposition.

### Data analysis

Data was captured in an Excel® (Microsoft Office, Microsoft Corporation, Redmond, WA) spreadsheet and checked for any errors or omissions. Data was analysed in the Statistical Package for the Social Sciences version 25. Most of the data was categorical and is reported descriptively as proportions and frequencies. Numerical data is reported as means and standard deviations or if not normally distributed, medians and interquartile ranges. The overall prevalence of trauma was calculated from the total number of entries in the register over the 4-month period and the number recorded as due to physical trauma. Children were defined as less than 13 years of age.

## Results

**Fig. 1:**
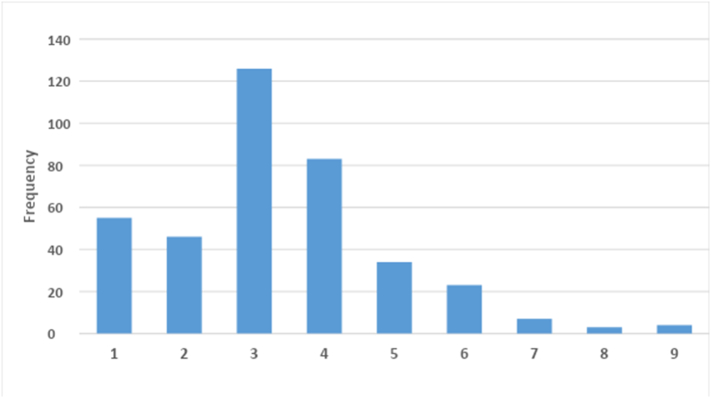
Age distribution of trauma cases.

A total of 14,873 patients attended the EC and 3657 sustained an injury or trauma, giving an overall proportion of 24.6%. Of these, 628 (17.1%) were young children less than 13-years of age and 3029 (82.9%) were adults or adolescents. Of these, 381 patient folders were sampled, 116 were female (30.4%) and 265 were male (69.6%), with mean age of 27.8 (SD 15.7) years. The mean age for females was 28.0 (SD 18.1) and males 27.7 (SD 14.6) years (Fig. 1).

Overall 74.5% of trauma patients came from three local communities (Nomzamo, Strand and Grabouw) and 61% of patients used the ambulance to access the EC (Table 1). The most frequent South African Triage Scale allocation was urgent (yellow) in 227 cases (59.6%), very urgent (orange) in 146 (38.6%), emergency (red) in 7 (1.8%) and non-urgent (green) in 1 (0.3%).

**Table 1:** Characteristics of trauma patients (N = 381).

Characteristics		n	%
Location	Gordon's Bay	6	1.6
	Grabouw	87	22.8
	Macassar	27	7.1
	Nomzamo	77	20.2
	Strand	120	31.5
	Somerset West	31	8.1
	Sir Lowry's Pass Village	16	4.2
	Other	17	4.5
Mode of transport to hospital	Ambulance	199	60.7
	Own Transport	126	33.1
	Other	3	0.8
	Missing data	53	13.9
SA triage system score on arrival	Green	1	0.3
	Yellow	227	59.6
	Orange	146	38.3
	Red	7	1.8
Disposition from emergency centre	Discharge	281	73.8
	Admit	39	10.2
	Transferred	49	13.3
	Unknown	12	3.1
Day of the week	Monday	57	15.0
	Tuesday	38	10.0
	Wednesday	39	10.2
	Thursday	40	10.5
	Friday	38	10.0
	Saturday	78	20.5
	Sunday	91	23.9

The majority of trauma cases were discharged (73.8%); 10.2% were admitted and 13.3% were transferred to tertiary hospitals. The weekend accounted for the bulk of trauma cases (44.3%), with Sunday being the busiest day and Monday also seeing a higher proportion of cases than other weekdays.

**Fig. 2:**
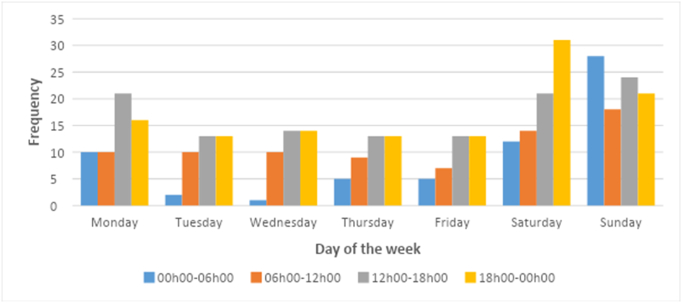
Frequency of trauma cases by time and day.

The busiest times for trauma cases were between 18 h00–24 h00 (31.8%) and 12 h00–18 h00 (31.2%) =, and the busiest period of the week was between 18 h00 on a Saturday until 06 h00 on a Sunday (Fig. 2).

As shown in Table 2, 49.1% of all trauma was accidental, including 13.1% from transport related injuries (Table 2). Intentional trauma, composed of community assaults, intimate partner and interpersonal violence accounted for 45.4% of all presentations.

**Table 2:** Non-intentional and intentional trauma by age group and sex (N = 381).

Type of trauma	Children N = 64 n (%)	Adults and adolescents N = 317 n (%)	Female N = 116 n (%)	Male N = 265 n (%)	Total N = 381 n (%)
Non-intentional					
Accidental	49 (76.5)	88(27.8)	50 (43.1)	87 (32.8)	137 (36.0)
Transport	9 (14.1)	41 (13.0)	23 (19.8)	27 (10.2)	50 (13.1)
**Total**	58 (90.6)	129 (40.8)	73 (62.9)	114 (43.0)	187 (49.1)

Intentional					
Community assault	0 (0.0)	13 (4.1)	0 (0.0)	13 (4.9)	13 (3.4)
Intimate partner violence	0 (0.0)	10 (3.2)	10 (8.6)	0 (0.0)	10 (2.6)
Interpersonal violence	1 (1.6)	149 (47.2)	22 (19.0)	128 (48.3)	150 (39.4)
**Total**	1 (1.6)	172 (54.4)	32 (27.6)	141 (53.2)	173 (45.4)

Unknown	5 (7.8)	15 (4.6)	11 (9.5)	10 (3.8)	21 (5.5)

Trauma in children was almost entirely accidental (90.6%), while most trauma in adults was intentional (54.4%). Likewise, trauma in women was mostly accidental (62.9%), while in men it was mostly intentional (53.2%). Amongst women, 31.3% of intentional trauma was due to intimate partner violence. Interpersonal trauma between men counted for more than a third of the total trauma burden (33.5%). Men were also responsible for all community assaults, although as with intimate partner violence, absolute numbers were fairly low.

In the 20–49-year age groups, the proportion of intentional trauma (including community assault, intimate partner violence and interpersonal violence) was higher than that of accidental trauma. Between ages 0–19 years and 60–89 years accidental trauma was more than intentional and at the extremes (0–9 years and 80–89 years) there was no intentional trauma recorded.

With regards to the disposition of different types of trauma, intentional trauma was responsible for a higher proportion of admissions compared to the accidental group (15.0% vs 6.5%). Transfers as a proportion was almost identical (13.1% vs 13.7%).

The most common mechanisms of injury were sharp injuries (27.6%), falls (22.0%), blunt trauma (19.4%) and transport-related (12.6%) (Table 3). Sharp injuries were defined as stabbing with a sharp object such as a knife, bicycle spoke, shard of glass or panga. Children under 13 years of age mostly suffered falls (46.9%), burns (15.6%) and motor vehicle or pedestrian injuries (12.5%). Adults on the other hand mostly presented with sharp injuries (31.2%), blunt force trauma (22.7%) and falls (17.0%).

**Table 3:** Mechanism of trauma by age, gender and disposition (N = 381). MVA = motor vehicle accident, PVA = pedestrian vehicle accident.

Mechanism of trauma	Children N = 64 n (%)	Adults and adolescents N = 317 n (%)	Female N = 116 n (%)	Male N = 265 n (%)	Admit N = 39 n (%)	Discharge N = 281 n (%)	Transfer N = 49 n (%)	Total N = 381 n (%)
Bite or sting	5 (7.8)	13 (4.1)	8 (6.9)	10 (3.8)	1 (2.6)	16 (5.7)	0 (0.0)	18 (4.7)
Blunt trauma	2 (3.1)	72 (22.7)	27 (23.3)	47 (17.7)	3 (7.7)	61 (21.7)	6 (12.2)	74 (19.4)
Crush injury	2 (3.1)	18 (5.7)	3 (2.6)	17 (6.4)	6 (15.4)	11 (3.9)	3 (6.1)	20 (5.2)
Fall	30 (46.9)	54 (17.0)	31 (26.7)	53 (20.0)	6 (15.4)	63 (22.4)	13 (26.5)	84 (22.0)
Burn/fire	10 (15.6)	8 (2.5)	9 (7.8)	9 (3.4)	4 (10.3)	12 (4.3)	2 (4.1)	18 (4.7)
Gunshot wound	0 (0.0)	9 (2.8)	4 (3.4)	5 (1.9)	0 (0.0)	3 (1.1)	6 (12.2)	9 (2.4)
MVA/PVA	8 (12.5)	40 (12.6)	22 (19.0)	26 (9.8)	2 (5.1)	38 (13.5)	6 (12.2)	48 (12.6)
Sharp	6 (9.4)	99 (31.2)	12 (10.3)	93 (35.1)	17 (43.6)	74 (26.3)	11 (22.4)	105 (27.6)
Other	1(1.6)	4 (1.3)	0 (0.0)	5 (1.9)	0 (0.0)	3 (1.1)	2 (4.1)	5 (1.3)

Of note, falls account for more than a quarter (26.5%) of all transfers to tertiary level hospitals, followed by sharp injuries (22.4%). Gunshot wounds, transport-related injuries and blunt trauma each accounted for a further 12.2% of transfers. As expected, 43.6% of all admissions were a result of sharp injuries. Crush injuries and falls each made up a further 15.4% of admissions.

## Discussion

A quarter of all patients in the EC presented with trauma at this urban district hospital. The majority were managed at the district hospital and only 13% needed transfer to a higher level of care. The proportion of trauma cases is similar to other South African district and secondary level hospitals, although Khayelitsha district hospital, in the same sub-structure, reported that 40% of their patients were trauma related [5]. This might be due to a larger catchment population with a lower socio-economic status and the ability to handle more complex trauma cases. Studies from neighbouring countries suggest that rates may be lower and could range from 21.6% of patients in northern Namibia to 3.5% in rural Malawi [17,22., [23], [24]]. Most of the patients were young men presenting with trauma on weekends and this pattern is consistently seen at all levels of the health system across South Africa [25,26].

A large proportion of this trauma related to inter-personal violence. Worldwide, it is estimated that intentional injuries account for 33% of the total trauma burden, significantly lower than the 45% in this study, potentially indicating higher levels of violence in South Africa compared to other places in the world [1,11]. Other studies in the Western Cape and Kwazulu-Natal highlighted the relationship between young men and intentional trauma [17,23]. Intentional trauma was commoner between 20 and 60 years and peaked in the 20–29 year age group. The morbidity and disability resulting from this trauma impacts working adults and therefore has substantial economic consequences for families [24].

Women were more likely to be injured in accidents, but were also more at risk of intimate partner violence. Up to 61% of women experience physical violence from an intimate partner [28] and although a third of all intentional violence against women in this study was from intimate partners, this is likely to be an underestimate of the real problem. Our findings do not include the number of women who do not present following assault [29] as well as other forms of abuse such as emotional and economic. In addition, the district has a separate service dealing with sexual assaults and therefore these patients will not always present to the EC. These women may also present to primary care facilities with non-specific symptoms which are difficult to attribute to intimate partner violence [27]. Nevertheless it is likely that staff working in the EC need to remain vigilant and proactive in recognising and assisting women with intimate partner violence [28].

The mechanisms of injury showed similar trends to other Western Cape studies [5,16]. Sharp injuries such as stabbings were the most common injuries, followed by blunt force, falls and motor vehicle or pedestrian accidents. The proportion of gunshot wounds was however low, compared to other studies [26]. Gunshot injuries in Cape Town are commoner due to accommodating gang-related violence on the Cape Flats [29].

The high proportion of sharp injuries may also be gang related, but in areas with high levels of crime and poverty, people tend to carry such weapons for self-defence. The use of knives in violence between known family and community members may also make a significant contribution. The rate of admission following intentional trauma was 15%, higher than the 5.2% reported elsewhere in Cape Town [17]. This may reflect a comparatively higher use of knives in this community, with penetrating chest injuries that required intercostal drains and hospitalisation, as opposed to assaults with blunt force trauma.

The majority of patients arrived by ambulance and only a third used their own transport. These patterns vary considerably between communities, as in neighbouring Khayelitsha use was more equal between own transport and ambulance [18], while in the city centre most patients were self-presenting [24]. Reasons for these different patterns may relate to the geographic spread of the catchment population. Helderberg Hospital is in a semi-rural community with much longer distances to travel that may make it difficult for poor communities to use their own transport. In addition, the hospital is not easily accessible by public transport. By contrast, the Khayelitsha Hospital serves a geographically dense and closer community with access by public transport. The utilisation of local ambulances to bring patients to Helderberg hospital may also impact on the availability of ambulances to transfer patients to higher levels of care and could potentially affect patient outcomes.

This study finds that men were the main perpetrators of community, family and interpersonal violence. Violence has become a normative and acceptable way of resolving conflict, and a way of asserting dominance within interpersonal relationships [1]. Although not evaluated in this study, others have highlighted the association between violence, young males, substance use, and weekends [6,30]. High inequality and significant unemployment also contribute to high levels of violence [9]. Although not investigated here, it is likely that harmful alcohol use, together with income inequality and unemployment are key underlying factors [6,9,16]. The recent alcohol ban as part of the national lockdown for the Corona virus pandemic resulted in an almost 60% reduction in assaults, accidents and other injuries and a 90% reduction in sexual assaults [31].

Violence is a complex problem and needs to be addressed in a comprehensive and holistic manner addressing infrastructure, healthcare and justice systems [32,33]. The WHO World Report on Violence and Health has made public health initiatives a priority to address the problem of violence [34]. While this study once again highlights the magnitude of violence in our community, this is just the first step. We also need to determine causal underlying pathways and identify feasible and effective interventions.

Crime and violence may have seasonal patterns, which would not be captured in this study, which focused on the summer months [34]. Violence may have a higher prevalence in the summer months making the results an overestimate of the real levels [34]. There were 42 trauma entries in the register without patient details, which could not be included. Cases with only the non-specific term “pain” might have been due to trauma, but were excluded. Patient records in temporary folders were difficult to locate, although only five selected folders could not be found and alternates were drawn. The mode of transport to the EC was not always reliably documented.

This hospital was typical of a moderate sized urban district hospital in the South African setting and while the results can only be generalised to this specific hospital the results would most likely be similar in other hospitals that share this context.

District hospitals play a substantial role in the definitive management of trauma. In the South African context, family physicians need to be well trained in the management of acute trauma resuscitation and stabilisation [35]. Most district hospitals will not have dedicated emergency medicine specialists. Clinical managers should ensure adequate staffing during the busiest times at weekends and that ECs are equipped and supplied to deal with the trauma burden.

The pressure on ambulance services for minor trauma might be relieved by access to 24-hour primary care in local communities. This might also reduce the overall number of minor cases presenting afterhours at the hospital. Accommodating access to primary care after hours remains a challenge in South Africa [36].

Health services should engage in a community-orientated primary care approach [37] to look at interventions to reduce trauma in the three most violent communities, for example, attention to law enforcement and sales of alcohol in informal and unregistered taverns. As part of this approach young males should be a target for prevention. Further research should investigate the underlying factors as well as feasible and effective interventions in the local context.

District hospitals, family physicians and other generalists are important in providing services for trauma in the South African context. High levels of intentional trauma, especially involving young males over the weekend, with high levels of sharp injuries were demonstrated. This trauma burden not only resulted in high numbers of admissions, but also placed a substantial workload on ambulances and emergency medical services. Further research is needed to identify underlying modifiable factors and to identify feasible and effective community-orientated interventions.

## Dissemination of results

Results were shared with the clinical manager and staff members at Helderberg Hospital. As the research was part of a Master's degree at Stellenbosch University the original assignment was also included in the open-access SunScholar repository of unpublished theses.

## Authors' contribution

Authors contributed as follow to the conception or design of the work; the acquisition, analysis, or interpretation of data for the work; and drafting the work or revising it critically for important intellectual content: TM contributed 80% and BM 20%. All authors approved the version to be published and agreed to be accountable for all aspects of the work.

## Declaration of competing interest

The authors declare no conflict of interest.
